# Translation, Adaptation, and Validation of the Brazilian Version of the Dickman Impulsivity Inventory (Br-DII)

**DOI:** 10.3389/fpsyg.2017.01992

**Published:** 2017-11-21

**Authors:** Áurea K. V. Gomes, Leandro F. M. Diniz, Guilherme M. Lage, Débora M. de Miranda, Jonas J. de Paula, Danielle Costa, Maicon R. Albuquerque

**Affiliations:** ^1^Postgraduate Program in Physical Education, Universidade Federal de Viçosa, Vicosa, Brazil; ^2^Department of Mental Health, Universidade Federal de Minas Gerais, Belo Horizonte, Brazil; ^3^Department of Physical Education, Universidade Federal de Minas Gerais, Belo Horizonte, Brazil; ^4^Department of Pediatrics, Universidade Federal de Minas Gerais, Belo Horizonte, Brazil; ^5^Department of Psychology, Faculdade de Ciências Médicas de Minas Gerais, Belo Horizonte, Brazil; ^6^Postgraduate Program in Molecular Medicine, Universidade Federal de Minas Gerais, Belo Horizonte, Brazil; ^7^Department of Sports, Universidade Federal de Minas Gerais, Belo Horizonte, Brazil

**Keywords:** dysfunctional impulsivity, functional impulsivity, cross-cultural adaptation, validation, factor analysis

## Abstract

Impulsivity has mainly been described as a negative or dysfunctional characteristic associated with several disorders. However, impulsivity is not only related to dysfunctional outcomes and may explain individual differences in optimal human functioning as well. The Dickman Impulsivity Inventory (DII) is a self-report instrument measuring both the dysfunctional and the functional aspects of impulsivity. In this study, we performed the translation and cultural adaptation of the DII to the Brazilian context and analyzed its psychometric properties. Translation and cultural adaptation followed a rigorous process, which relied on an expert panel in the cross-cultural adaptation of psychological instruments. Data from 405 undergraduate students were obtained for the Brazilian version of the DII (Br-DII). The 23 items of the Br-DII was considered unsuitable according to model fit indices of the Confirmatory Factor Analysis (both for Oblique and Orthogonal models). Exploratory Factor Analysis showed an 18 items version of the Br-DII to be suitable (CFI = 0.92; TLI = 0.90, and RMSEA = 0.057). The DII’s 18 items version also showed adequate Cronbach’s alpha, intraclass correlation coefficient, and convergent and discriminant validity with the BIS-11. Therefore, the Br-DII demonstrated reliability and validity in the measurement of functional and dysfunctional impulsivity.

## Introduction

Maladaptive Impulsive Behavior, which can be defined as a “predisposition toward rapid unplanned reactions to internal or external stimuli without regard to the negative consequences of these reactions to themselves or others” (Moeller et al., 2001, p. 1784). Maladaptive expression of impulsivity is often observed in psychiatric disorders such as attention-deficit/hyperactivity disorder (Malloy-Diniz et al., 2007; Dang et al., 2014) antisocial personality disorder (Swann et al., 2009), borderline personality disorder (Cackowski et al., 2014), affective disorders (Peters et al., 2015), and substance abuse and addiction (Gray and MacKillop, 2015). Since most studies of impulsivity are rather focusing on its undesirable, dysfunctional consequences (Malloy-Diniz et al., 2007; Lage et al., 2013; Lin and Zhang, 2015; Nederkoorn et al., 2015). Thus, the instruments designed to measure impulsivity are biased toward its negative outcomes. The well-known *Barratt Impulsiveness Scale* (BIS) (Patton et al., 1995) and the *Behavioral Inhibition System*/*Behavioral Activation System* (BIS/BAS) (Carver and White, 1994) are examples of the focus only on the dysfunctional consequences of impulsivity.

Using a broader impulsivity concept that would be defined as a predisposition to quickly, non-planned reactions despite their consequences (Moeller et al., 2001). Assuming that impulsivity is a complex trait and most likely does not represent a unitary construct (Dalley et al., 2011), and that as many psychological traits, impulsivity is not only related to dysfunctional outcomes and may explain individual differences in optimal human functioning as well (Pluess and Belsky, 2013). Dickman (1990) suggests that impulsivity can have functional outcomes for simple and well-structured tasks, for example, where rapid responses are advantageous despite errors (Dickman, 1985). The tendency to act rapidly and with relatively little forethought can be useful in a context where time is very restricted to one’s decision or movement (Dickman and Meyer, 1988; Lage et al., 2012). Impulsivity can be functional, for example, in open skills sports such as soccer, basketball, and handball. In these sports, due to the constant changes in the environment, players are forced to inhibit pre-planned responses, anticipate actions and coordinate body segments based on the complex and dynamic flow of sensory information (Lage et al., 2011) with little time available (Raab, 2012). However, despite the concept of functional impulsivity being used in the sports and motor control fields (Lage et al., 2011, 2012), and that the concept of impulsiveness is broader than the concept of maladaptive impulsive behavior, no study so far used a specific measure of functional impulsivity to test the role of impulsivity in optimal human functioning in those areas. In the Brazilian context, this may be due to the lack of an adapted and validated an instrument to measure functional impulsivity.

The Dickman’s Impulsivity Inventory (DII) subdivides the construct impulsivity into two subtypes: dysfunctional impulsivity and functional impulsivity (Dickman, 1990). Dysfunctional Impulsivity is the tendency to make quick decisions and act with less forethought when this tendency is non-optimal or a source of difficulty. Functional Impulsivity is the tendency to make quick decisions and act with little forethought when it is optimal and beneficial. The DII is the only instrument that evaluates the functional dimension of impulsivity and has been used in several investigations (e. g., Roozen et al., 2013; Fino et al., 2014).

The DII was translated and adapted for several languages other than the original English (Guillemin et al., 1993; Widenfelt et al., 2005). The DII is currently available in Dutch (Claes et al., 2000), French (Caci et al., 2003), Spanish (Chico et al., 2003), Chinese (Gao et al., 2011), and Italian (Leone et al., 2002).

In summary, although there is no gold standard for the translation, adaptation, and validation of a measure cross-culturally, the literature agree that merely translating a scale in itself inadequate. In the present manuscript, we chose to use consolidated methods to translate, adapt and validate the DII, by factor analysis, Cronbach’s alpha, Intraclass Correlation Coefficient (ICC), and for divergent and convergent validity by BIS-11. Therefore, in this study, we aimed to translate, adapt, and validate the DII for the Brazilian context.

## Materials and Methods

### Cross-Cultural Adaptation

**FIGURE 1:**
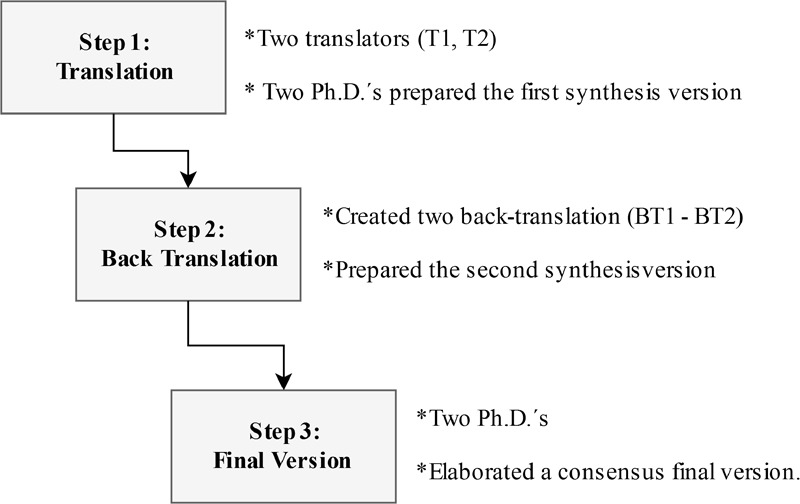
Summary of the Cross-cultural adaptation method.

The cross-cultural translation and adaptation were done following the methods proposed by Guillemin et al. (1993), Beaton et al. (2000), and Hambleton et al. (2005). The cross-cultural adaptation (**Figure 1**) started by the translation of the original Dickman’s Impulsivity Inventory (Dickman, 1990) to the Brazilian Portuguese by two native Brazilian Portuguese speakers fluent in English. The translations were done separately, producing two independent Brazilian versions (T1 and T2). Two Ph.D. researchers with experience in translation, adaptation, and validation of scales, and Impulsivity compared the different translations and evaluated any semantic discrepancies (including any linguistic or conceptual issues). After these translation comparisons, a merge and synthesis of the two versions was obtained. The synthesis (T1 – T2) version was independently back-translated to English (BT1 and BT2) by two English native speakers fluent in Brazilian Portuguese that whom later they met and produced a synthesis version. Original and back-translated versions were reviewed, compared and adjusted for equivalence by consensus among the same two Ph.D. researchers and the linguist. After conducting a small pilot study with five subjects to improve the understanding of the items, the same two Ph.D. researchers and the linguist compared the back translation synthesis and prepared the final version of the Brazilian version of the Dickman’s Impulsivity Inventory (Br-DII).

### Validation

#### Sample

According to Gudmundsson (2009), an instrument must be administered to a fairly large sample to be accurately adapted. Our convenience sample was composed of 405 undergraduate students (217 male and 186 female) with a mean age of 22.90 (±4.31) years. Our sample size follows the recommendations of Brown (2006) and Kline (2011) for EFA and CFA of a ration of 10:1 (ratio of the number of the subjects per number of items). To evaluate the consistency of the measure (reliability) by the test-retest method, 83 subjects (20.49%) of the sample responded to DII 2 weeks after the first application of the scale.

Regarding economic classification, the sample was distributed as follows: high – 250 (62%); middle – 135 (33%) and low – 20 (5%). The study was approved by the local ethical committee of the Federal University of Viçosa (protocol number: 44032415.9.0000.5153), and all participants signed an informed consent after receiving a full explanation of the study.

#### Instruments

The *Dickman’s Impulsivity Inventory* (Dickman, 1990) is composed of 23 self-report items, from which 11 were designed to measure functional impulsivity and 12 to measure dysfunctional impulsivity. The original version published by Dickman (1990) has a dichotomous (True-False) response format. However, we adopted a Five-point (Totally disagree, Disagree, Neutral, Agree and Totally agree) ordinal response scale (Leone et al., 2002) since Likert-type instruments show higher sensibility (Capik and Gozum, 2015).

To investigate both convergent and discriminant validity of the DII, the Brazilian version of the *Barratt Impulsiveness Scale* (BIS-11) was used (Malloy-Diniz et al., 2010). BIS-11 is a 30-item, four-point ordinal, and a self-report instrument developed to assess motor impulsivity, non-planning impulsivity, and inhibitory control. However, in the present study, we used the total score of the scale, a more global measure of impulsivity (Malloy-Diniz et al., 2015). In summary, higher scores in the BIS-11 indicate higher impulsivity. The BIS-11 total score presented a Cronbach’s alpha coefficient of 0.81 in this study.

Participants’ socioeconomic status was assessed by using the Brazilian Criteria of Economic Classification (CCEB). The CCEB is a 9-item questionnaire that assesses the available resources at home and the educational level of the household. The CCEB total score ranges from 0 to 46 points. As used in previous research (Costa et al., 2016), these economic classes can be divided into three classes: “high” (A and B classes; median monthly household income from U$2349 to U$4152); “middle”(C class; median monthly household income from U$514 to U$1190); and “low”(D and E classes; median monthly household income of U$348).

#### Data Analysis

Confirmatory (CFA) and Exploratory Factor Analysis (EFA) were used to assess the construct validity of the Brazilian version of the DII in this study. EFA was done by using the Geomin oblique rotation method, and both CFA and EFA were performed with weighted least squares (WLSMV) estimator due to the ordinal nature of the scale (e.g., a Likert-type scale of fewer than seven points) (Chen et al., 2015). These analyses were done to test the DII two-factorial structure: functional impulsivity and dysfunctional impulsivity.

A range of indices was used to assess how well the data fit the proposed model. These indices were the chi-square value and corresponding *p*-value, the relative chi-square statistic, the root means square error of approximation (RMSEA), the comparative fit index (CFI), and the Tucker-Lewis Index (TLI). Widely adopted guidelines are available to gauge how well a model fits the data. Concerning the χ^2^/df index, a value of less than 2 indicates a good fit. A RMSEA value of 0.08 or lower also indicates that a model can be considered adequate to fit the data. A CFI and TLI with a value of 0.90 can be considered as adequately fitting the data (Brown, 2006; Kline, 2011).

Cronbach’s alpha was calculated to assess Br-DII’s internal consistency, and the Pearson correlation coefficient between the BIS-11 and the Br-DII was performed to measure its convergent and discriminant validities. Test-retest reliability was assessed by the Intraclass Correlation Coefficient (ICC), and paired t-tests were run to compare the responses within a 2 weeks interval.

In agreement analysis between 23-items and 18-items, the scores were calculated by the mean of the values of each item of the factors (Functional and Dysfunctional). The agreement analysis between 23-items and 18-items scores separated by factors was analyzed using Bland and Altman method (Bland and Altman, 1986). Bland and Altman (1986) purposed the method to quantify the agreement between two quantitative measurements by limits of agreement. These limits are calculated by using the mean and the standard deviation of the differences between two measurements. In general, a plot is used to show the results from the Bland and Altman method.

Bland and Altman method analysis was conducted using Medcalc (version 12.5). CFA and EFA were run in the Mplus, version 6.12. SPSS version 20.0 was used to conduct all other analysis.

## Results

### Translation and Cross-Cultural Adaptation

**Figure 1** shows the step-by-step of the translation and cross-cultural adaptation of the DII to the Brazilian context; including the final Brazilian version approved by the expert panel (see **Table 1**).

**Table 1:** Translation and cross-cultural adaptation of *Dickman’s Impulsivity Inventory.*

	Original version	Brazilian version
1	Often, I don’t spend enough time thinking over a situation before I act	Na maioria das vezes, eu não gasto muito tempo pensando sobre uma situação antes de agir.
2	I try to avoid activities where you have to act without much time to think first.	Eu tento evitar atividades nas quais tenho que agir sem muito tempo para pensar antes.
3	I don’t like to make decisions quickly, even simple decisions, such as choosing what to wear, or what to have for dinner.	Eu não gosto de tomar decisões rapidamente, mesmo decisões simples, como escolher o que vestir ou o que comer no jantar.
4	I enjoy working out problems slowly and carefully.	Eu gosto de resolver problemas de forma lenta e cuidadosa.
5	I am good at taking advantage of unexpected opportunities, where you have to do something immediately or lose your chance.	Eu sou bom em aproveitar oportunidades inesperadas, em que você tem que fazer algo imediatamente, ou perde sua chance.
6	I would enjoy working at a job that required me to make a lot of split-second decisions.	Eu gostaria de trabalhar em um emprego que me exigisse tomar várias decisões em frações de segundo.
7	I often make up my mind without taking the time to consider the situation from all angles.	Eu frequentemente tomo decisões sem gastar tempo analisando a situação de todos os ângulos.
8	I have often missed out on opportunities because I couldn’t make up my mind fast enough.	Muitas vezes perco oportunidades porque eu não consigo decidir com rapidez suficiente.
9	I often say and do things without considering the consequences.	Eu frequentemente digo e faço coisas sem levar em conta as consequências.
10	I frequently make appointments without thinking about whether I will be able to keep them.	Eu frequentemente marco compromissos sem pensar se vou ser capaz de cumpri-los.
11	I am uncomfortable when I have to make up my mind rapidly.	Eu me sinto desconfortável quando tenho que tomar decisões rápidas
12	I don’t like to do things quickly, even when I am doing something that is not very difficult.	Eu não gosto de fazer coisas rapidamente, mesmo quando eu estou fazendo algo que não é muito difícil.
13	I frequently buy things without thinking about whether or not I can really afford them.	Eu frequentemente compro coisas sem pensar se realmente posso ou não adquiri-las.
14	I’m good at careful reasoning.	Eu sou bom em raciocinar cuidadosamente.
15	I like to take part in really fast-paced conversations, where you don’t have much time to think before you speak.	Eu gosto de participar de conversas rápidas, nas quais você não tem muito tempo para pensar antes de falar.
16	I like sports and games in which you have to choose your next move very quickly.	Eu gosto de esportes e jogos nos quais você tem que decidir o próximo movimento muito rapidamente.
17	Many times the plans I make don’t work out because I haven’t gone over them carefully enough in advance.	Muitas vezes os planos que faço não funcionam porque eu não os examinei suficientemente com cuidado em antecedência.
18	I often get into trouble because I don’t think before I act.	Eu costumo ter problemas por não pensar antes de agir.
19	Most of the time, I can put my thoughts into words very rapidly.	Na maioria das vezes, eu posso transformar rapidamente meus pensamentos em palavras.
20	People have admired me because I can think quickly.	As pessoas me admiram por eu pensar rapidamente.
21	I will often say whatever comes into my head without thinking first.	Eu costumo dizer as coisas que vêm a minha mente sem pensar antes.
22	Before making any important decision, I carefully weigh the pros and cons.	Antes de tomar qualquer decisão importante eu peso cuidadosamente nos prós e contras.
23	I rarely get involved in projects without first considering the potential problems.	Eu raramente me envolvo em projetos sem primeiro considerar os possíveis problemas.

### Confirmatory Factor Analysis (CFA)

The two-dimensional structure (Dysfunctional and Functional) of the 23 Br-DII’s items was tested through CFA. CFA evaluated the fit of the oblique and orthogonal model for the two-factor solution. The fit of the 23 items of the Br-DII was considered not suitable considering both indices (**Table 2**).

**Table 2:** Fit indices for Confirmatory Factor Analysis Models.

	χ^2^	Gl	χ^2^/gl	CFI	TLI	RMSEA	Results
Oblique	1101.893^∗^	229	4.81	0.74	0.71	0.097 (0.091-0.103)	Unsuitable
Orthogonal	965.259^∗^	230	4.19	0.78	0.76	0.089 (0.083-0.095)	Unsuitable

*CFI, Comparative Fit Index; TLI, Tucker-Lewis Index; RMSEA, Root Mean Square Error of Approximation; ^∗^*p* < 0.05.*

### Exploratory Factor Analysis (EFA)

As shown in **Table 3**, an EFA suggested that the two-dimensional structure (Dysfunctional and Functional) of the 23 Br-DII’s items is not suitable (see EFA1).

**Table 3:** Fit indices for Exploratory Factor Analysis of the Brazilian version of DII.

	Number of items	Deleted items	χ^2^	Gl	χ^2^/gl	CFI	TLI	RMSEA	Results
EFA1	23	–	551.303	208	2.65	0.90	0.88	0.064 (0.057-0.070)	Unsuitable
EFA2	20	4, 7, and 15	426.105	151	2.82	0.88	0.85	0.067 (0.060-0.075)	Unsuitable
EFA3	18	4, 7, 9, 10, and 15	274.981	118	2.33	0.92	0.90	0.057 (0.049-0.066)	Suitable

*EFA, Exploratory Factor Analysis; CFI, Comparative Fit Index; TLI, Tucker-Lewis Index.*

Based on the Factor Loading, three items were excluded in the next analysis (EFA2). Item 4 was excluded for showing a large loading value in the Functional Impulsivity factor, whereas it was considered a Dysfunctional Impulsivity item in the original DII version. Item 7 also showed a loading value above 0.30 in a different dimension than that proposed by the original DII version, and item 15 did not show factor loading value above 0.30 in any of the DII’s impulsivity dimensions. Therefore, the second EFA (EFA2) was conducted with 20 items (**Table 3**). Again, the 20 items version of the Br-DII had fit indices considered not suitable.

**FIGURE 2:**
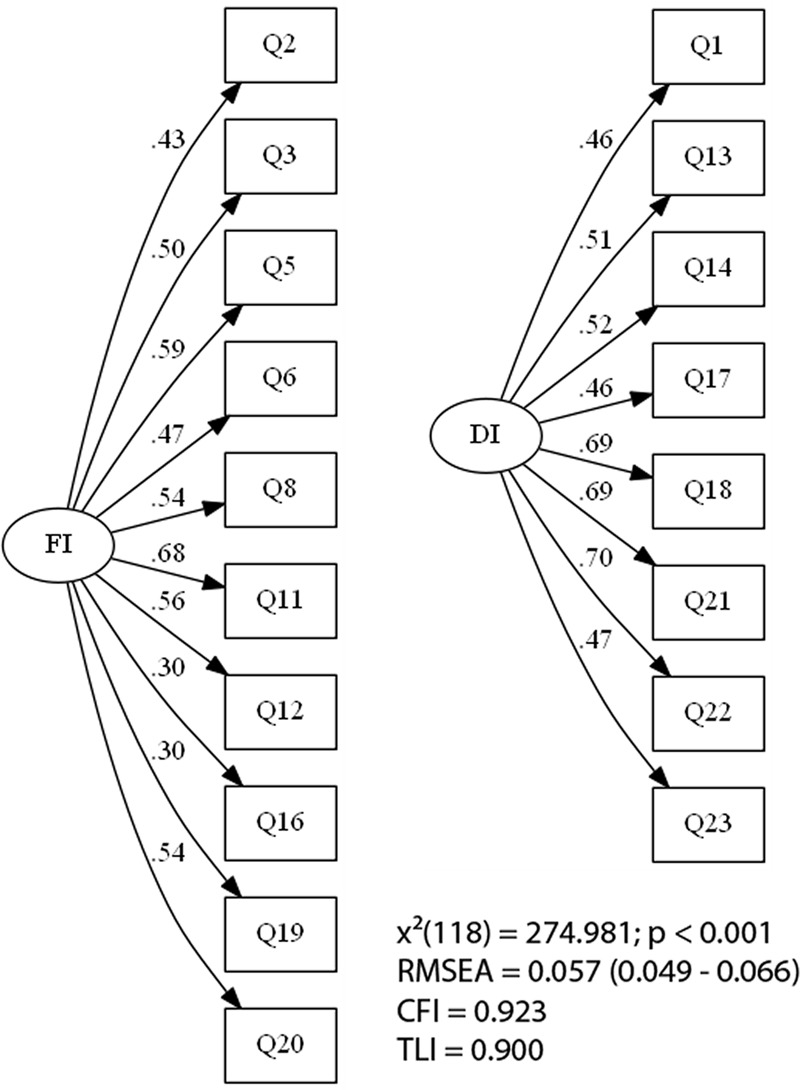
Fit indices for Exploratory Factor Analysis with Factor Loads; FI, Functional Impulsivity; DI, Dysfunctional Impulsivity; RMSEA, Root Mean Square Error of Approximation; CFI, Comparative Fit Index; TLI, Tucker-Lewis Index.

In a third model, two more items (items 9 and 10) were excluded for being somewhat different to the structure found in the original DII version. These items showed a loading value above 0.30 in a different dimension of the one suggested in the original version of the scale. The third and last EFA (EFA3) was conducted with 18 items (**Table 3**). The final version of the Br-DII had eight items loading in the Dysfunctional dimension and 10 items in the Functional dimension. The 18 items of the DII’s Brazilian version presented excellent fit indices (see **Figure 2**).

### Reliability

The Br-DII internal consistency, which was considered sufficient. The Cronbach’s alpha of the 23-items version was 0.81 for the Dysfunctional dimension and 0.74 for the Functional dimension. The reliability of the 18-items version was considered adequate with Cronbach’s alpha of 0.75 and 0.73 for the Dysfunctional and Functional dimensions, respectively.

Scores in the Functional Impulsivity domain did not change significantly over a 2-weeks period [*t*(82) = 1.321; *p* = 0.190] with a mean of 2.97 (±0.58) in the first measurement and 2.91 (±0.60) in the last measurement. The ICC was 0.89 (*p* < 0.001) indicating good test-retest stability. Cronbach’s alpha was 0.70 in the first measurement and 0.76 in the second measurement. Regarding the Dysfunctional Impulsivity domain, no significant changes between the first and second measurements were found observing a 2-weeks period [t(82) = 0.541; *p* = 0.590)]. A mean of 2.35 (±0.60) was observed for the first measurement and a mean of 2.33 (±0.59) after the 2-weeks interval. The ICC was 0.85 (*p* < .001) indicating good test–retest stability. Cronbach’s alpha was 0.71 in the first measurement and 0.74 in the second measurement.

### Convergent and Discriminant Validity

Our results showed that the two DII dimensions, Functional and Dysfunctional Impulsivity, seem to be independent of one another (*r* = -0.031; *p* = 0.539). We also observed the correlation between the Functional and the Dysfunctional Impulsivity DII scores with the Barratt Impulsiveness Scale (BIS-11). BIS-11 showed a significant and positive association with the Dysfunctional Impulsivity dimension (*r* = 0.633; *p* < 0.001), but not with the Functional Impulsivity dimension (*r* = -0.035; *p* = 0.483).

### Agreement between of Factor Scores

**FIGURE 3:**
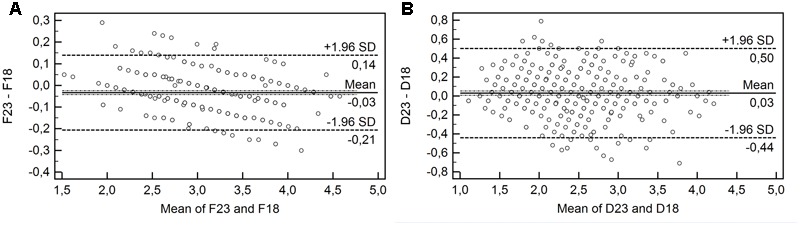
Bland-Altman plots. The dashed bold lines represent the mean difference score. The dashed lines represent the limits of agreement (mean ± 1.96 × the standard deviation of the difference score); **(A)** Functional Impulsivity and **(B)** Dysfunctional Impulsivity.

Bland and Altman plots of data from 18-items and 23-items versions are shown in **Figure 3**. In Functional [mean bias of -0.03 lower (-0.21) and upper (0.14) 95% confidence interval] and Dysfunctional [mean bias of 0.03 lower (-0.44) and upper (0.50) 95% confidence interval] factors produced a low mean bias. In addition, 18-items version are significant and positively association with 23-items in Functional (*r* = 0.988; *p* < 0.001) and Dysfunctional (*r* = 0.924; *p* < 0.001).

### Interpretative Parameters of the 18-Items and 23-Items Versions of the Dickman’s Impulsivity Inventory for the Brazilian Context (Br-DII)

**Table 4** shows the normative data for the Functional and the Dysfunctional Impulsivity DII scores in the full sample.

**Table 4:** Interpretative parameters of the 18-items and 23-items versions.

	*D*_18_	*D*_23_	*F*_18_	*F*_23_
Mean	2.41	2.44	3.13	3.09
*SD*	0.62	0.60	0.57	0.55
Cronbach’s alpha	0.75	0.81	0.73	0.74
Percentile 5	1.50	1.52	2.20	2.20
Percentile 25	2.00	2.00	2.70	2.72
Percentile 50	2.37	2.33	3.10	3.09
Percentile 75	2.75	2.83	3.60	3.45
Percentile 95	3.62	3.58	4.00	3.97
Percentile 99	4.12	4.07	4.49	4.45

*D_*18*_, Dysfunctional with 18 items; *D*_*23*_, Dysfunctional with 23 items; *F*_*18*_, Functional with 18 items; F_*23*_, Functional with 23 items; *SD*, standard deviation. Higher scores in the functional domain are indicative of a higher functional impulsivity; Higher scores in the dysfunctional domain are indicative of a higher dysfunctional impulsivity.*

## Discussion

This study aimed to translate, adapt, and validate the DII for the Brazilian context (Br-DII). Translation and cultural adaptation followed a rigorous process, which relied on an expert panel in the cross-cultural adaptation of psychological instruments. We found that an 18 items version rather than the original 23 items version of the DII was suitable for use in the Brazilian population. Although Br-DII has fewer items than the original version (Dickman, 1990), our results showed that the final Brazilian version demonstrates suitable model fit. Also, Cronbach’s alpha, intraclass correlation coefficient, convergent and discriminant validity confirmed the quality of our version of the scale.

In general, most of the self-report scales were developed in English-speaking countries, but cross-cultural and international collaborative studies are needed to researchers of non-English speaking countries to have access to reliable and valid instruments (Beaton et al., 2000). Nowadays, there are well-established methodological approaches for translating, adapting, and validating instruments (e.g., Beaton et al., 2000; Sperber, 2004). However, there is no clear consensus on how these approaches should be used (Maneesriwongul and Dixon, 2004). The *Dickman Impulsivity Inventory* (DII) was previously adapted and validated for some languages such as Spanish (Chico et al., 2003), French (Caci et al., 2003), Chinese (Gao et al., 2011), Italian (Leone et al., 2002), and Dutch (Claes et al., 2000). Notwithstanding, the translation process used were heterogeneous with some being more or less rigorous (Chico et al., 2003; e.g., Claes et al., 2000; Leone et al., 2002). However, none of them reported the use of well-defined methodological approaches to guide the translation (Claes et al., 2000; Leone et al., 2002; Caci et al., 2003; Chico et al., 2003; Gao et al., 2011). Thus, to the best of our knowledge, our study used the most rigorous approach, when compared to the other translations of the DII.

The original version of the scale proposed by Dickman (1990) was developed with answers in the dichotomous (true/false) format. Nevertheless, in the Br-DII version, we used a five-point Likert scale format (Totally disagree, Disagree, Neutral, Agree, and Totally agree). Although dichotomous scales do not allow misunderstandings, since the answers are straightforward (For example, Yes/No or True/False), the Likert scale seems to be a more sensitive measure when compared to the dichotomous format (Preston and Colman, 2000). So, in reading the question, the individual selects the most appropriate response within a range of options (Zou et al., 2010; Capik and Gozum, 2015). Also, some studies have shown that adapting scales from the dichotomous format to the Likert scale format can often improve internal consistency and validity (e.g., Preston and Colman, 2000; Capik and Gozum, 2015; Finn et al., 2015). Among the studies of adaptation of the DII, the Italian version of Leone et al. (2002) also used the Likert scale of five points.

Since the DII should measure distinct aspects of impulsivity, the dysfunctional and functional dimensions of the DII should exhibit relatively low or none correlation between them. In some DII versions, this relationship is endorsed. For example, in the Italian version a *r* < 0.30 was found (Leone et al., 2002); *r* = 0.32 in the Spanish version (Chico et al., 2003), *r* = 0.23 in the French version (Caci et al., 2003), *r* = 0.23 in the American version (Dickman, 1990), *r* = 0.25 in the Chinese version (Gao et al., 2011), and *r* = -0.02 in the Dutch version (Claes et al., 2000). A very low and non-significant association was also found in Jones and Paulhus (2011) study. In the present Brazilian study, we also observed an independence between the Functional and Dysfunctional factors with an *r* = -0.03.

Different statistical approaches have been used to validate instruments. In summary, EFA and CFA can be used. The previous DII’s validation studies used EFA but did not assess model fit (Claes et al., 2000; Caci et al., 2003; Chico et al., 2003; Gao et al., 2011). Model fit is obtained through several statistical tests used to determine how well a model fits the data and is considered a robust statistical technique to validate scales (Brown, 2006; Kline, 2011). Despite evidence of low or no association, there is no consensus regarding the correlation between the DII’s functional and dysfunctional dimensions (Claes et al., 2000; Caci et al., 2003; Chico et al., 2003; Gao et al., 2011). Thus we tested two models in the CFA. First, we used the oblique model, which allows for the correlation between the factors (Brown, 2006). Secondly, we tested the orthogonal model, which forces the solution to reach uncorrelated latent variables (Brown, 2006). In both CFA models tested, the adjustment indicators did not reach the cut-off points established in the literature of CFI and TLI (≥0.95), and RMSEA (<0.07) (Brown, 2006; Kline, 2011, 2013). Therefore, we sought to identify the Br-DII factor structure through EFA.

The first EFA showed that three items (i.e., 4, 7, and 15) behaved differently from that of the original version (Dickman, 1990). We then performed the second EFA without those three items (4, 7, and 15) and found another two items (9 and 10) showing factor loading above 0.30 in a dimension different from that of the original proposal (Dickman, 1990). The third and last EFA was run excluding six items (i.e., 4, 7, 9, 10, and 15) showing a suitable fit in the model. The final version of the Br-DII had all parameters recommended for a good model fit [χ^2^_(118)_ = 274.981; *p* < 0.001; CFI = 0.92, TLI = 0.90, RMSEA = 0.057], and all items behaved similarly to the original version (Dickman, 1990). When verifying the agreement between the two versions (18-items and 23-items) of the scale, it was possible to identify a small bias between the two versions, as well as a high correlation between the two scores of the versions. This fact can be related to the use of the mean for the final calculation of the score that even without the totality of the items being computed the mean was able to keep values close and highly correlated.

To test convergent and discriminant validity, we used the well-documented Barratt Impulsiveness Scale (BIS-11) (Malloy-Diniz et al., 2010), which focuses on the dysfunctional aspect of impulsivity. As expected, the BIS-11 had a strong correlation with the DII’s Dysfunctional dimension, while presenting a low and non-significant association with the DII’s Functional dimension. Cronbach’s alpha is an important measure of the internal consistency reliability (Field, 2009). For the Dysfunctional and Functional DII’s dimensions, respectively, the Cronbach’s alphas were 0.76 and 0.78 in the Spanish version (Chico et al., 2003); in the Italian version were 0.78 and 0.75 (Leone et al., 2002), in the French version were 0.79 and 0.75 (Caci et al., 2003), in the American version were 0.85 and 0.74 (Dickman, 1990), in the Chinese version Cronbach’s alphas for the control group were 0.75 and 0.68 (Gao et al., 2011), in the Dutch version were 0.85 and 0.84 (Claes et al., 2000). Our study also showed appropriate Cronbach’s alpha values (i.e., 0.75 and 0.73 for Dysfunctional and Functional impulsivity, respectively). The test-retest reliability assessment showed that the responses were stable over the 2-weeks period. The Intraclass Correlation Coefficient (ICC) was high for both DII’s dimensions (dysfunctional and functional) indicating good test-retest reliability (Cicchetti and Sparrow, 1981). Overall, the Br-DII showed good psychometric properties. However, studies with larger and more representative samples should be conducted in the future to ensure the maintenance of these qualities.

This study presents some limitations. First, we did not control for the presence of psychiatric disorders in our sample. Such control would allow us to verify if the instrument would be sensitive to identify patients with psychiatric disorders since dysfunctional impulsivity is a hallmark of several mental disorders (Moeller et al., 2001; Cackowski et al., 2014; Gray and MacKillop, 2015). Also, it would be possible to understand the role of functional impulsivity in this context. Another limitation concerns the characteristics of the sample that was composed of undergraduate students, which is not representative of the overall Brazilian population. The level of schooling is an important variable for several psychological scales and may influence their validity and reliability (Caldas et al., 2012). In the Brazilian context, education level is highly heterogeneous, although the younger generations had a substantial increase in school years. Most instruments have been developed in countries where educational levels are high, which can produce false-positive results when those instruments are applied to populations with low educational levels (De Yebenes et al., 2003). Therefore, we are aware that a more heterogeneous sample is recommended for testing the applicability of the DII in the Brazilian context.

Finally, it is possible to verify Dickman’s (1985, 1990) assumptions in an ecological context: (1) If subjects with higher functional impulsivity score respond better to relatively simple but time constraint tasks? (Dickman, 1985, 1990; Lage et al., 2012) (2) If there is an association between kinematic analysis and impulsivity in motor control? (Lage et al., 2012), and (3) If years of practices in different types of sports (with and without time restrictions) present different functional impulsivity scores (Lage et al., 2011). Therefore, the present study will make possible new investigations on the functional impulsivity topic.

In summary, the Dickman’s Impulsivity Inventory (DII) proposed by Dickman (1990) was translated and adapted to the Brazilian Portuguese language. The present study evaluated the psychometric properties of the Br-DII. The analyses suggested that the DII adaptation is valid and reliable for use in Brazilian samples.

## Author Contributions

Conceived and designed the experiments: AG, LD, GL, and MA. Collected Data: AG and MA; Analyzed the data: AG, JdP, DdM, DC, and MA. Wrote the paper: AG, LD, GL, DdM, JdP, DC, and MA.

## Conflict of Interest Statement

The authors declare that the research was conducted in the absence of any commercial or financial relationships that could be construed as a potential conflict of interest.
